# Modernising public health surveillance in an EHDS-enabled Europe: building a coordinated European public health intelligence ecosystem

**DOI:** 10.3389/fpubh.2026.1974886

**Published:** 2026-10-19

**Authors:** Miguel Ferreira-Pinto, Nuno P. Silva, Sara Silva Marques, Nuno Cruz, Eva Gouveia Anjo, Cátia S. Pinto

**Affiliations:** Global Digital Health and International Affairs Unit, SPMS - Shared Services of the Ministry of Health, E. P. E., Lisbon, Portugal

**Keywords:** European Health Data Space (EHDS), HealthData@EU, secondary use of health data, public health surveillance, emergency preparedness, public health intelligence ecosystem

## Abstract

**Background:**

Public health surveillance in Europe is facing increasingly complex cross-border threats. Although digital health investments and EU-level initiatives have expanded the amount of health data available for surveillance purposes, the generation of timely and actionable public health intelligence remains constrained by disease-specific reporting systems and systemic technical and governance barriers. The European Health Data Space (EHDS) Regulation provides a new opportunity for the use and exchange of routinely electronic health data to improve public health surveillance and emergency preparedness, but its effective contribution remains insufficiently explored.

**Objective:**

Through a scoping literature review and regulatory analysis, this study examines the role of the EHDS in strengthening public health surveillance and emergency preparedness in Europe.

**Methods:**

This review followed the PRISMA-ScR guidelines. Multiple bibliographic databases were searched for studies published between 2016 and 2026, complemented by an analysis of grey regulatory literature. Eligible studies included publications addressing the EHDS or secondary use of health data and public health surveillance or emergency preparedness.

**Results:**

The scoping review included 35 scientific studies and six grey literature documents. Our findings suggest that health system digitalisation, Electronic Health Records (EHR) implementation and the COVID-19 pandemic have reshaped surveillance and preparedness needs in Europe, which remain limited by fragmented infrastructures, distributed health data, limited interoperability, and unclear legal and governance mechanisms. Our regulatory analysis indicates that the EHDS could help address several of these challenges by establishing common governance rules, secure processing conditions, and federated access mechanisms. We further propose a conceptual European public health intelligence ecosystem, positioning the EHDS as an enabling layer for converting distributed health data into actionable intelligence. Three non-exclusive governance options illustrate how this model could be operationalised across national and European levels.

**Conclusions:**

This study establishes the EHDS as an important enabling framework for modernising public health surveillance in Europe. The conceptual framework proposed in this study establishes a foundation for future research on the development of an EHDS-enabled European public health intelligence ecosystem to strengthen surveillance, preparedness, and coordinated emergency response. Future EU initiatives and implementation pilots will be essential to test, validate, and translate this vision into practice.

## 1 Introduction

Public health surveillance systems are undergoing a profound transformation. The increasing frequency and complexity of public health threats, including infectious disease outbreaks, antimicrobial resistance, climate-related health emergencies, environmental hazards, and the increasing prevalence of chronic diseases, call for the modernisation of existing public health surveillance systems (1). Recent global health crises, particularly the SARS-CoV-2 (COVID-19) pandemic, further exposed limitations in surveillance, preparedness, and health data use across Europe (2). According to the World Health Organization (WHO), the COVID-19 pandemic was responsible for approximately 7.0 million officially reported deaths and associated with around 22.1 million excess deaths globally between 2020 and 2023 (3). Therefore, beyond its direct health impact, the pandemic exposed vulnerabilities and limitations across public health systems, generating disruptions to healthcare delivery, delayed diagnosis and treatment, economic instability, and broader societal challenges (4, 5).

In response, Europe reinforced its public health surveillance and preparedness architecture. The role of the European Center for Disease Prevention and Control (ECDC) was strengthened through an updated strategic long-term surveillance framework for the European Union (EU)/European Economic Area (EEA), aiming to improve epidemic intelligence, disease reporting, early detection of cross-border infectious disease threats, and coordinated public health responses between Member States (6). In parallel, the Health Emergency Preparedness and Response Authority (HERA) was established to reinforce the EU capacity to coordinate preparedness and response activities to large-scale cross-border health threats. HERA aims to foster cooperation between Member States, support the centralised purchasing of medical countermeasures (MCM), and enable the rapid activation of emergency measures (7). Although these initiatives were relevant for strengthening infectious disease surveillance and preparedness, existing surveillance infrastructures often remain organised around disease-specific reporting systems, which may limit their capacity to integrate broader health data sources and support more comprehensive public health intelligence.

Aiming to improve public health intelligence, the European Commission prioritised the digital transformation of health systems, contributing to the widespread adoption of digital technologies and electronic health records (EHR) (8, 9). This has generated unprecedented volumes of routinely collected health data that can be reused for other purposes, creating new opportunities to strengthen surveillance, improve preparedness, and support evidence-informed public health decision-making (10, 11). As such, European authorities must be able to generate public health intelligence from both traditional epidemiological reporting systems and diverse complementary data sources, including EHR, medical imaging repositories, genomic sequencing, and non-traditional sources such as mobility data (11, 12). This broader data foundation could strengthen public health surveillance and emergency preparedness by supporting earlier disease detection, better resource allocation, improved situational awareness, and more informed public health action (13). However, the ability to access, use and share distributed health data in a timely, trustworthy, and actionable manner to improve public health surveillance and emergency preparedness remains challenging due to technical and governance persistent barriers (14). As European health systems face persistent workforce shortages, demographic changes, and increasing pressure to deliver data-driven public health responses, overcoming these structural barriers must be a strategic priority (15, 16).

In this context, the European Health Data Space (EHDS) Regulation (EU) 2025/327 was introduced as a regulatory and governance framework to improve the use and exchange of electronic health data for diverse purposes across Europe (17). The EHDS represents an important opportunity to support more structured, secure, and interoperable access to health data, while enabling federated and privacy-preserving approaches that respect data protection requirements and national data governance responsibilities (17, 18). Therefore, understanding how the EHDS can contribute to generate actionable public health intelligence from diverse data sources is necessary to maximise its potential contribution to European health threat detection, surveillance, preparedness, and response.

Several publications discuss the EHDS and its role for research and innovation, but comparatively fewer studies examine how it could support public health surveillance and emergency preparedness (19). In this study, we address this gap by mapping the existing evidence on how digital health transformation, the implementation of EHR and the COVID-19 pandemic have shaped health data use for public health surveillance and emergency preparedness in Europe and by identifying the persistent barriers that continue to limit its effective use and exchange. We then examine the potential role of the EHDS as an enabling layer for complementing existing surveillance systems with distributed health data and how its governance mechanisms and secondary use infrastructure could be leveraged to strengthen European surveillance and preparedness. Relevant use cases are further analysed to illustrate how these mechanisms could help address the identified challenges by supporting more secure, interoperable, and trusted use of distributed health data. Drawing on the findings of both analyses, we propose an EHDS-enabled European public health intelligence ecosystem and three non-exclusive governance options to illustrate how this model could be operationalised at national and European levels.

## 2 Methods

A scoping review was conducted to identify opportunities and challenges for an effective public health surveillance and emergency preparedness in Europe and to explore how the EHDS, particularly through its secondary-use mechanisms, could support the development of a more coordinated and resilient European public health intelligence ecosystem. A scoping review approach was considered appropriate because the relationship between the EHDS, secondary use of health data, public health surveillance, and emergency preparedness remains an emerging and cross-disciplinary field, involving scientific literature, regulatory provisions, policy documents, and implementation examples.

The review followed the PRISMA-ScR guidelines and was designed to identify and synthesise existing evidence on EHDS-related mechanisms relevant to public health surveillance and emergency preparedness, including digital health tools, EHR implementation, secondary use of health data, and health data governance. This was complemented by a regulatory and policy analysis of the EHDS framework to examine how its provisions could respond to the opportunities and challenges identified in the literature.

### 2.1 Literature search strategy and regulatory analysis

A literature search was conducted to identify scientific publications relevant to the EHDS, secondary use of health data, public health surveillance and emergency preparedness. The search covered publications from January 2016 to May 2026. This timeframe was selected to capture developments before, during, and after the COVID-19 pandemic, since it reflected a period of substantial technological, regulatory, and organisational change in digital health, public health surveillance, and health data governance.

The electronic databases PubMed/MEDLINE, ScienceDirect, IEEE Xplore and Web of Science were searched using combinations of keywords related to public health surveillance, emergency preparedness, and the European Health Data Space. The Boolean search expression was:

(“European Health Data Space” OR “EHDS” OR “secondary use of health data” OR “Health Data Space”) AND (“surveillance” OR “preparedness”).

For each database, the same Boolean expression was used, while searching methods were adapted according to each database's search functionality and indexing system.

Google Scholar was used as a supplementary source to identify potentially relevant publications that may not have been captured through the primary bibliographic database searches. In line with methodological recommendations for evidence synthesis, screening was limited to the first 200 records ranked by relevance (20). We acknowledge both the added value of Google Scholar for complementary searching and its limitations related to proprietary ranking algorithms and decreasing relevance of lower-ranked results.

To complement the scientific literature, targeted searches of regulatory and policy documents were performed through EUR-Lex and the websites of relevant European and international institutions and EU-funded initiatives, including the ECDC, WHO, Organisation for Economic Co-operation and Development (OECD) and the TEHDAS2 and EUCAIM EU projects. These sources were consulted to identify legislation, strategic frameworks, policy documents, technical guidance, and official reports relevant to secondary use of health data under the EHDS, public health surveillance, and emergency preparedness.

### 2.2 Study selection and eligibility criteria

The identification, screening, eligibility assessment, and inclusion of scientific studies followed a selection process consistent with PRISMA-ScR guidance (21) (Figure 1). A total of 427 records were identified through the bibliographic database search. Records were exported from each database and duplicates were removed before screening. Publications not written in English and non-open access articles were also excluded at this stage. Titles and abstracts were then screened for relevance by two reviewers independently. Disagreements were resolved through discussion and, when necessary, consultation with another author.

**Figure 1:**
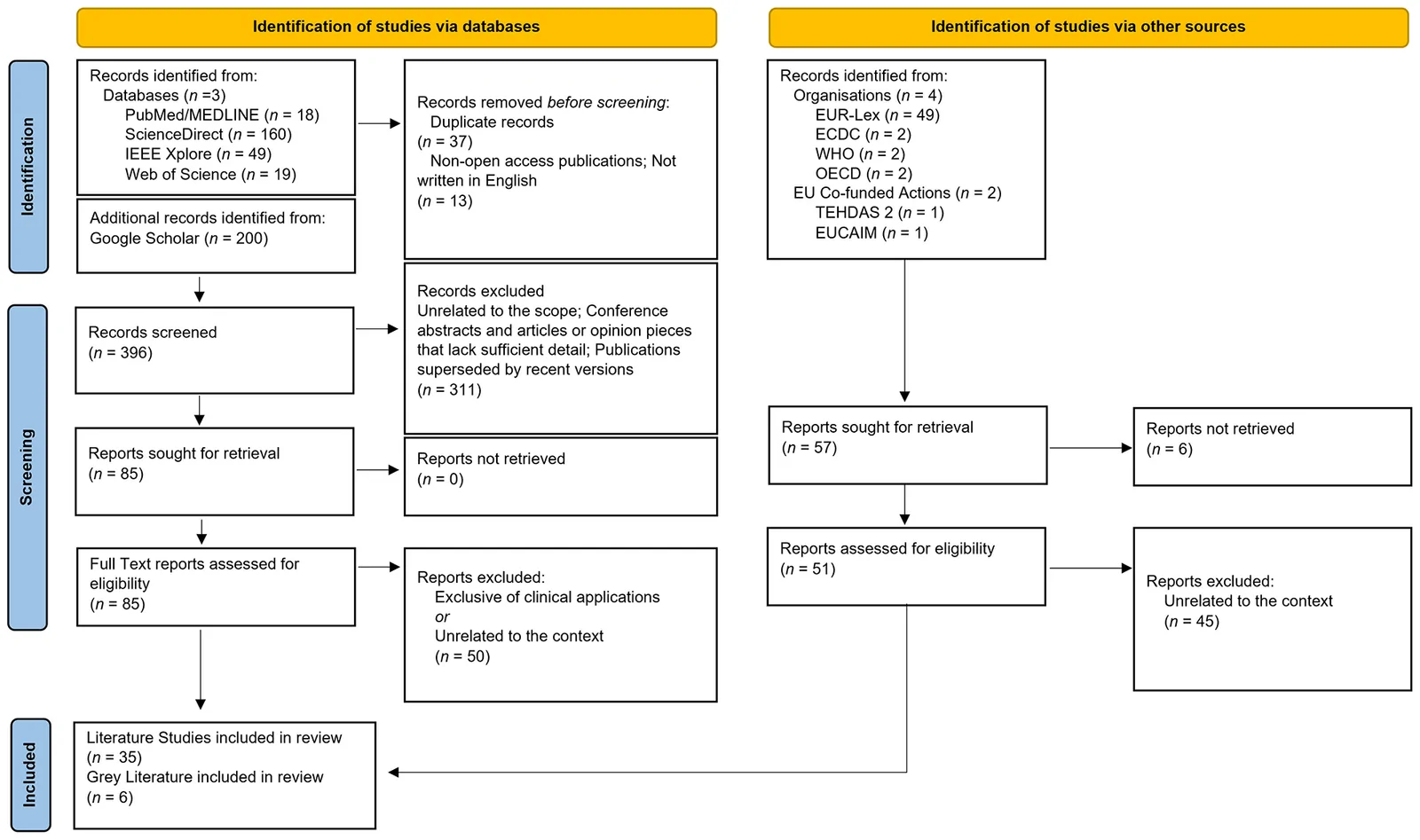
PRISMA 2020 flow diagram of data collection and analysis, showing the included literature based on the inclusion and exclusion criteria.

Eligible studies included peer-reviewed research articles, review articles, policy analyses, conceptual frameworks, case studies, pilot studies, and other scientific publications addressing the EHDS or secondary use of health data in relation to public health surveillance, emergency preparedness, or crisis response. Studies describing European initiatives were also included when their findings were relevant to strengthening surveillance or preparedness capacities. At this stage, studies were excluded if they focused exclusively on clinical applications without addressing public health surveillance, emergency preparedness, or the secondary use of health data; if they described public health initiatives unrelated to the European context or to the scope of the study; or if they were conference abstracts without sufficient detail, articles or opinion pieces without substantive methodological, empirical, or conceptual contributions, or publications superseded by more recent versions.

Full texts of potentially eligible publications were assessed for their relevance to the secondary use of health data under the EHDS, public health surveillance, and emergency preparedness. Following full-text assessment, 35 scientific studies and six grey literature documents were included in the scoping review.

### 2.3 Data extraction, synthesis, and development of the conceptual model

Data were extracted from the included studies according to their relevance to the research objective. The included articles addressed the EHDS, secondary use of health data, interoperability requirements, health data governance, digital health infrastructures, EHR implementation and the COVID-19 pandemic in relation to public health surveillance, emergency preparedness, or crisis response. Two reviewers independently conducted data extraction, with disagreements resolved through discussion.

Findings were synthesised across two analytical domains. The first examined opportunities and challenges for strengthening European public health surveillance and emergency preparedness, with particular attention to health system digitalisation, EHR implementation, secondary use of health data, and lessons learned from the COVID-19 pandemic. The second analysed EHDS provisions, implementation examples and relevant use cases to assess how the Regulation could complement existing surveillance systems and support the development of a European public health intelligence ecosystem. Articles discussing the EHDS implementation opportunities and challenges were also synthesised, presented in the Results section and then extensively discussed in section 5.4 of the Discussion.

## 3 Results

The scoping review included 35 scientific studies and six grey literature, regulatory, and policy documents. Section 3.1 specifically addresses how health system digitalisation and the COVID-19 pandemic have created both opportunities and challenges for European public health surveillance and emergency preparedness evolution. Section 3.2 examines how the EHDS regulatory framework, together with selected use cases, could help address these challenges and support the development of a European public health intelligence ecosystem.

### 3.1 Digital health transformation, EHR implementation and COVID-19: opportunities and challenges of health data use in public health surveillance and emergency preparedness

The growing digitalisation of health systems and EHR implementation have expanded the potential of health data to support public health surveillance and emergency preparedness (12, 22). Digital public health tools can improve the efficiency and effectiveness of public health action by supporting health data use for disease prevention, surveillance, risk factor management, patient engagement, and e-health literacy, while also depending on high data quality and robust technical, legal, organisational, and interoperable infrastructures (23). The evolution of digital epidemiology further suggests that diverse digital data sources can strengthen disease monitoring and public health intelligence, provided that data collection, analysis, and sharing follow robust standards, avoid bias, ensure metadata transparency, and allow comparability across countries (12). Despite these opportunities, the adoption of digital health solutions has not progressed uniformly across Europe. Although e-health use increased across EU countries between 2016 and 2022, persistent disparities in healthcare digitalisation remain between countries and population groups, creating additional barriers for vulnerable and disadvantaged populations (24). National public health systems have also adopted digital solutions in different ways (25). This illustrates the diversity of approaches through which digital public health can support the collection and analysis of large volumes of health data to identify public health trends and enable more targeted interventions, but also reinforced the need to ensure equitable access across populations with different levels of digital access.

In parallel, the widespread implementation of EHR has created new opportunities for the secondary use of routinely collected healthcare data. Although originally designed to support direct care, EHR are increasingly recognised as resources for public health surveillance, epidemiological research, health system management, and emergency preparedness (17). EHR data can support disease preparedness through clinical research, disease prediction, MCM allocation and research, and enabling more proactive monitoring of community health status, which could provide near-real-time insights into the health status of communities and support the automatic tracking of disease patterns (13). Within this context, the creation of comprehensive health data ecosystems integrating EHR, genomic data, wearable devices and mobile health applications data, internet-based platforms, cloud computing, and artificial intelligence (AI) tools can improve disease detection, outbreak forecasting, disease patterns and population health trends monitoring, and public health decision-making, provided that data quality, findable, accessible, interoperable and reusable (FAIR) principles, ethical and regulatory frameworks, and cross-sectoral collaboration are ensured (26).

This vision is aligned with the ECDC, which recognised the potential of routinely collected healthcare data for infectious disease surveillance (22). The ECDC Long-Term Surveillance Strategy 2021–2027 calls for harmonised surveillance systems, optimal combination of data sources, and digital technologies to generate continuous, automated, integrated, and, where appropriate, real-time data streams for public health action (6). Importantly, the use of EHR for surveillance can also extend beyond infectious disease outbreaks to include non-communicable diseases, chronic conditions, risk factors, mental health, injuries, and environmental and occupational risks (27). Despite these opportunities, European health information systems remain affected by fragmented data sources, limited accessibility, restricted data use and reuse, and difficulties in EHR implementation (28). Moreover, sustainable cross-border cooperation also remains limited by differences in health systems, administrative structures, legal frameworks, and local conditions, reinforcing the need for greater harmonisation of health information systems to support secure EHR exchange and more integrated cross-border public health activities (29).

The COVID-19 pandemic made these limitations particularly visible and marked a turning point in the European debate on digital maturity and health data sharing. It accelerated collaboration between the European Commission, Member States, researchers, and other stakeholders, and led to increased investment in data-sharing projects and coordinated initiatives, such as the European COVID-19 Data Platform (2). Initiatives such as ReCoDID (*Reconciliation of Cohort data in Infectious Diseases*), ORCHESTRA (*Connecting European Cohorts to Increase Common and Effective Response to SARS-CoV-2 Pandemic*), unCoVer (*development of an efficient steganalysis framework for uncovering hidden data in digital media*), and SYNCHROS (*Synergies for Cohorts in Health: integrating the Role of all Stakeholders*) sought to identify and address these limitations through data harmonisation, cohort linkage, and cross-border data exchange, but further efforts were identified as necessary to improve common standards and the legal, technical, and organisational conditions for health data sharing (2). Indeed, lessons learned from the ReCoDID project, which tested the linkage between laboratory and clinical-epidemiological data using a centralised approach, highlighted significant delays in data handling related to GDPR (General Data Protection Regulation (EU) 2016/679) compliance requirements, limited FAIR alignment, difficulties in data access approvals, and resource-intensive processes of using a centralised approach (30).

Other studies reinforced that these limitations were not restricted to individual datasets or specific projects, but reflected broader structural barriers to data use for surveillance and during health emergencies (31–33). Limited data standardisation restricted the reuse of routinely collected healthcare data for surveillance, research, and emergency response, highlighting the need for stronger international coordination by strengthening the leadership role of WHO, improved equity in access to technology, and greater collaboration in the use of health data for preparedness (31). Indeed, when COVID-19 monitoring across 31 European countries was attempted, limitations related with inconsistent recording, restricted public availability, and poor comparability of primary healthcare indicators were found, reinforcing the need for common indicators, uniform disease coding systems, interoperability standards, and systematic data collection processes to support secondary use of data for disease surveillance (32). In the context of COVID-19 vaccination, real-world evidence supported rapid public health decision-making on dosing regimens, target populations, booster strategies, vaccine safety, and responses to emerging variants, but data use was limited by poor data quality, standardisation and availability, privacy requirements, regulatory transparency gaps, and uneven infrastructure and expertise across countries (33).

As seen in the ReCoDID project, centralised approaches can be feasible for linking distributed data during public health emergencies, but important practical limitations, particularly the time-consuming and resource-intensive nature of handling and storing clinical data centrally were also highlighted (30). This led to the growing interest in decentralised and federated approaches, which may reduce the need for large-scale data transfer and central storage by allowing data to remain within national or institutional environments while enabling authorised analysis across distributed data holders (30, 34). However, important gaps remain in the operational use of data federation during emergencies, particularly regarding the legal exchange of sensitive data and trusted collaboration among EU emergency agencies (34). This highlights the need for common EU-level rules, federated information-sharing systems, and standardised mechanisms for exchanging information during public health emergencies.

Beyond traditional healthcare data, non-traditional data sources, such as mobility data, social media traces, satellite imagery, and other digital streams, may be important to complement public health data during crises, but their use depends not only on better access, harmonisation, quality, and standardisation, but also on data solidarity, literacy, and translating regulation into actionable policy interventions (35). As such, the technical and operational requirements to improve surveillance and preparedness are also closely linked with ethical and legal governance challenges that continue to limit health data use across Member States (35, 36). Cross-border health data sharing during emergencies remains limited by privacy protection, data-sharing agreements, political accountability, fragmented legal frameworks, unclear institutional responsibilities, and the need for trusted repositories and data access committees' actions (37). A more streamlined framework, guided by solidarity, trust, transparency, accountability, fairness, and reciprocity, could therefore support more timely and equitable data sharing during public health emergencies (37). As such, research ethics and governance frameworks should be further refined to place greater emphasis on preparedness, international collaboration, flexible ethical review, research prioritisation, and responsible data sharing for future public health crises (36).

Indeed, the importance of addressing these challenges was recognised at EU policy level. The Council of the European Union reflected on the COVID-19 lessons learned and called for increased digital maturity of healthcare systems, improved infrastructure interoperability, greater standardisation of health records, and the development of a common European health data space (38). These developments contributed to a broader transformation of EU health policy, reinforcing mandates for the ECDC and the European Medicines Agency (EMA), supporting the creation of HERA, and prioritising the EHDS as a central initiative to strengthen health data use, exchange, and governance in Europe (8, 39). As such, the post-pandemic evolution of European health governance has been marked by a decisive shift from reactive crisis management towards a more anticipatory and integrated approach aimed at strengthening institutional resilience and preparedness (39).

The following section examines how the EHDS Regulation regulatory framework can enable the secure and governed secondary use of health data to improve public health surveillance and emergency preparedness.

### 3.2 The EHDS as an enabling governance framework for public health surveillance and emergency preparedness

The EHDS Regulation emerges as a central regulatory response to address some of the existing challenges to public health systems, establishing a common European framework for the use and exchange of electronic health data, and enabling the secure, trustworthy, transparent and timely access to health-related data (17). By providing harmonised rules for applying to data access, data sharing, interoperability, and governance, the EHDS seeks to reduce data fragmentation and supports data use in accordance with existing EU data protection regulations, including the GDPR, Data Governance Act (DGA) Regulation (EU) 2022/868 and the Data Act Regulation (EU) 2023/2854.

The relevance of the EHDS for surveillance and preparedness is explicit in the Regulation. Recital 1, which frames the aim of the Regulation, identifies health threats preparedness and response, including preventing and addressing future pandemics, among the societal purposes that can benefit from electronic health data. Recital 2 further states that COVID-19 demonstrated the importance of timely access to quality electronic health data for efficient surveillance and better management of future pandemics. These principles are operationalised in Article 53, which establishes permitted purposes for processing electronic health data for secondary use, including in the areas of public or occupational health, such as activities to protect against serious cross-border threats to health, public health surveillance, among other purposes as scientific research, health policymaking, and regulatory activities. Therefore, the EHDS provides a legal basis for using health data beyond primary care, explicitly recognising surveillance and emergency preparedness as a legitimate secondary use purpose.

Article 51 defines the minimum categories of electronic health data that may be made available for secondary use, including EHR data; determinants of health; aggregated data on healthcare needs, resources, access, expenditure, and financing; pathogen data; population-based registries; and health data from biobanks and associated databases. Besides establishing relevant purposes and data categories, the EHDS Regulation defines the institutional and infrastructural building blocks through which secondary use can be operationalised. It offers an opportunity to create a flexible, unified and interconnected federated system that preserves citizen control and privacy while also supporting the primary and secondary use and exchange of health data (40). As such, the Regulation foresees an interconnected governance structure involving health data holders, health data access bodies (HDABs), health data intermediation entities, health data users, secure processing environments, and HealthData@EU.

Health data holders are defined as the entities with the legal right or obligation to process personal electronic health-related data, as well as those responsible for making non-personal health-related data available. As controllers of these datasets, health data holders are mandated to provide their data to the HDAB upon an approved data application. HDABs, the central governance actors in the EHDS infrastructure, are responsible for managing access to electronic health data in an efficient, secure, harmonised, and transparent way. Their tasks include making available public dataset catalogues, assessing and deciding on data access applications and data requests, issuing data permits, and ensuring that approved access occurs under the conditions defined by the Regulation. The EHDS also allows Member States to provide for health data intermediation entities, which can assist data holders by processing, registering, making available, and exchanging health data on their behalf.

The framework and structure posed by the EHDS for secondary use are particularly relevant, replacing fragmented, bilateral, and *ad hoc* data-sharing arrangements with a legally supervised pathway for discovering, requesting, accessing, and analysing health data across EU Member States. HealthData@EU, the common European infrastructure for the secondary use of electronic health data, operationalises this federated and decentralised approach by providing a central platform connecting national HDABs, the Union health data access service, and authorised participants through a common infrastructure for dataset discovery and cross-border applications (Figure 2). National contact points for secondary use act as technical gateways between national infrastructures and the HealthData@EU platform, enabling metadata from national catalogues to be communicated to the European catalogue and cross-border applications to be transmitted to the relevant HDABs. Interestingly, to safeguard urgent public health needs, Article 68 requires shorter assessment timeframes for applications submitted by public sector bodies for epidemiological surveillance, emergency preparedness and response. In addition, although opt-out rights apply to secondary use, Article 71 allows national opt-out exceptions for certain public health purposes when the use of the requested data is necessary, proportionate, and no suitable alternative exists. Together, these provisions show that the EHDS is designed not only to support research, innovation, and policymaking, but also to enable more timely, legally supervised, and trusted access to health data when evidence is needed to detect, monitor, and respond to public health threats (17, 41). In this sense, the EHDS can function as an enabling layer for a more integrated European public health intelligence ecosystem, creating the governance, interoperability, and secure access conditions needed to complement existing surveillance systems and strengthen emergency preparedness across Member States.

**Figure 2:**
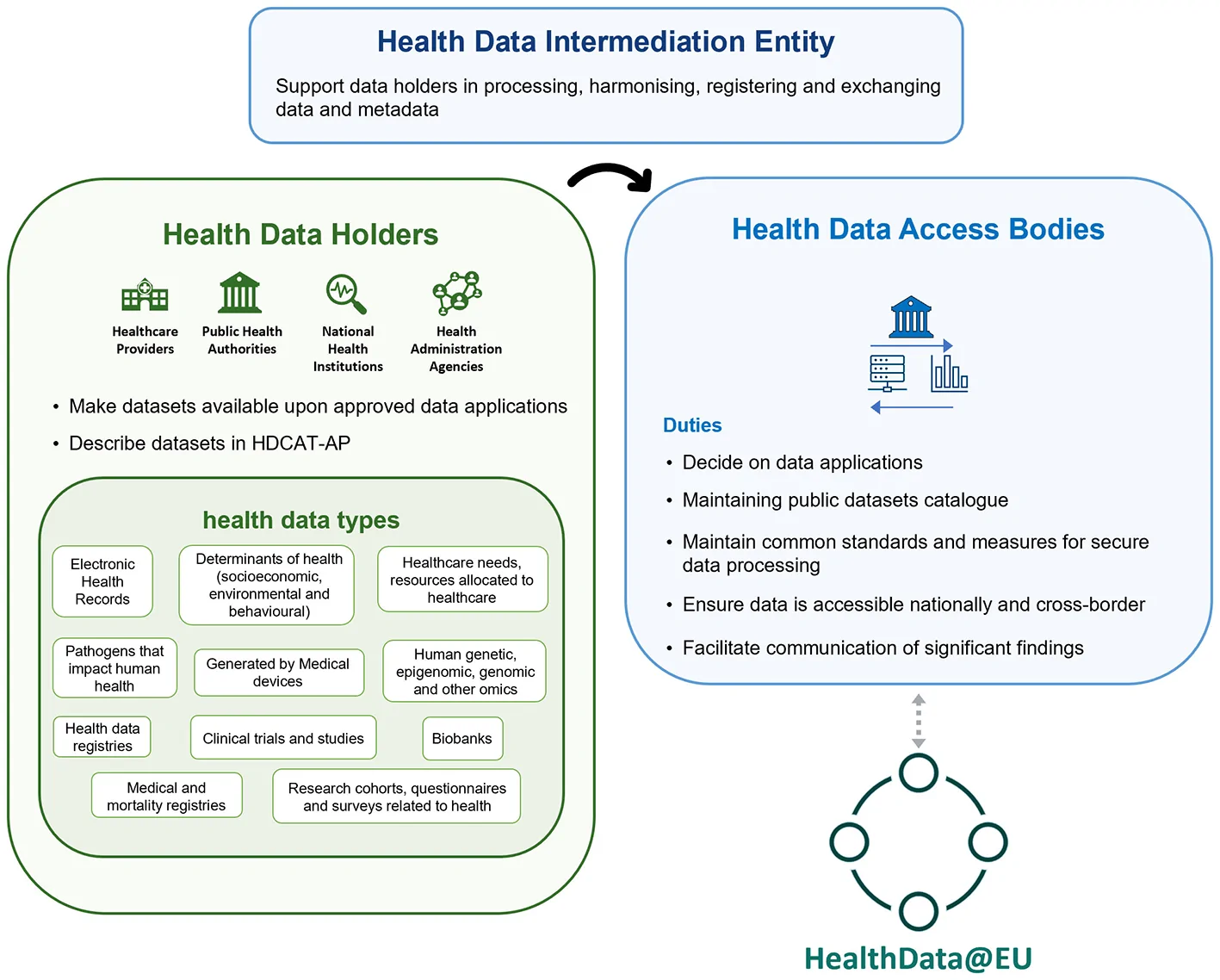
Scheme illustrating the governance framework proposed in the EHDS Regulation for data use and sharing. Data Holders make available categories of data (Article 51), including those relevant to public health surveillance. Datasets are described in a Health DCAT Application Profile (HDCAT-AP) standard format and made available if a data request is approved (Article 60). Metadata and data both flow to the HDAB, which has an obligation to maintain a public platform to facilitate finding these datasets (Articles 57, 58). A health data intermediation entity can assist health data holder in this process (Article 50). The dataset descriptions (metadata) in this public catalogue flow into the EU Central Services (HealthData@EU) to facilitate EU-wide data discovery.

#### 3.2.1 EHDS-enabled data use for public health intelligence: selected use cases

The literature review also identified several use cases and implementation examples that illustrate how the EHDS regulatory provisions could be operationalised to enable health data access and exchange for public health surveillance and emergency preparedness. These examples show how dispersed health data can be transformed into public health intelligence through federated infrastructures, secure processing environments, common data models, interoperability standards, and cross-border governance mechanisms. Rather than representing a single implementation pathway, they provide practical insights into the technical, legal, organisational, and governance conditions needed to make EHDS-enabled data use to gather actionable public health intelligence for surveillance, preparedness, and response.

A study by Schneider et al. (42) piloted the HealthData@EU infrastructure function through two use cases led by EMA and the ECDC, focusing on coagulopathy-related events in COVID-19 patients and antimicrobial resistance surveillance, respectively. The pilot tested the potential of HealthData@EU to support dataset discovery, data access applications, and data use by EU-level public health and regulatory bodies. The study highlighted the important contribution of HealthData@EU to improve data discoverability and accessibility, but it also revealed important implementation gaps. The EMA use case suggested that the use of observational medical outcomes partnership (OMOP) mapping improved interoperability. However, the limited experience of certain national nodes in OMOP mapping, the variability in national application requirements, and long assessment timeframes were key challenges to the practical implementation of this use case. The ECDC use case relied on a distributed approach, requiring data processing and transfer agreements between the participating data-holding nodes and ECDC. The procedure showed high concordance between the AMR case counts generated through the transferred analysis package and the historical values published in the EARS-Net AMR report. However, this process was found time-consuming and complex as it required adaptation to national legal contexts and interpretations regarding data protection. Due to this complexity and lack of a standardised cooperation framework, decisions on data applications took approximately 1 year to be issued. Both use cases highlighted the need of harmonised requirements and timeframes for data application assessment as well as the importance of standardised procedures for data application in the EU. This study demonstrated the potential of the EHDS secondary use infrastructure to support communicable disease surveillance and real-world data use by organisations such as EMA and ECDC, provided that challenges related to data fragmentation, national application requirements, and country-specific legal interpretations are overcome.

Other initiatives provide complementary lessons on the technical and organisational capacities needed to make the secondary use infrastructure operational closer to the data source. León-García et al. (43) developed a hybrid federated regional data space for secondary use built using open-source components. In this model, data remained locally held, while a federated central node was responsible for terminology mapping, OMOP conversion, de-identification, cataloguing, data quality improvement, and privacy-preserving operations, partially resembling the HDAB infrastructure foreseen in the EHDS Regulation. This approach illustrates that secondary use infrastructures require technical preparation and strong data governance procedures that foster accountability and clearly identify roles and duties among data holders, HDAB, and data users. Essential components for the operationalisation of these data exchange infrastructures, included data harmonisation processes to address the heterogeneity of EHR and clinical terminologies, pipelines for improving data quality, and the implementation of federated systems, all of which necessitate in-house expertise, and large infrastructure and harmonisation investments. This example demonstrates the technical requirements needed to support HDAB-mediated access, secure processing, and interoperable secondary use under the EHDS framework.

The EUropean Federation for CAncer Images (EUCAIM) project illustrates a pan-European federated infrastructure for oncological imaging data access and analysis, supporting cancer research and the development of AI-driven cancer solutions (44). EUCAIM's model connects distributed cancer imaging data nodes to a central hub while preserving institutional data sovereignty. Through common data models, semantic and metadata standards, federated querying, distributed processing, and privacy-preserving operations, EUCAIM offers a practical example of the federated data-sharing model envisioned by the EHDS for streamlined and efficient health data exchange.

Other studies further illustrate how decentralised approaches aligned with the capabilities of the EHDS infrastructure could enable more specialised forms of public health intelligence gathering. For example, ethically governed federated systems have been proposed as a way to connect intensive care units (ICU) across Europe, transforming fragmented ICU data into shared intelligence for sepsis research and personalised critical care (45). Automated healthcare-associated infection surveillance has also been considered feasible in Europe, although unevenly implemented since it requires adequate digital infrastructure, validated protocols, data quality monitoring, interoperability, IT support, and training. In this context, the large-scale implementation of this system may benefit from the EHDS to improve data interoperability and support more harmonised rules for secondary use of health data (46). Similarly, van Rooden et al. (47) explored the application of federated systems specifically to automated infection surveillance. They described a model in which centrally developed algorithms can be applied locally by healthcare facilities, supporting methodological standardisation, local data control, privacy preservation, reduced reporting burden, cross-country comparability, and near-real-time data access. In the proposed model, source data is extracted locally from EHR systems and, depending on the surveillance objective and governance arrangements, case-based data, aggregated data, or analytical outputs can then be shared at regional, national, or international level, while the underlying source data remain under local control.

Several studies also examined opportunities and implementation challenges related to EHDS-enabled data use for public health surveillance and emergency preparedness, such as Europe-wide coordinated data governance (48), the advantages and limitations of federated approaches (49, 50), opportunities for non-communicable disease monitoring (51) and One Health surveillance (52–54), and AI-supported public health intelligence (55, 56). A deeper discussion of these studies can be found in Section 5, sub-section 5.2.

Together, the regulatory analysis and implementation examples suggest that the EHDS could help Europe move from fragmented health data systems towards a more coordinated public health intelligence ecosystem. Its added value lies in increasing access to data and creating the conditions for governed data usability through harmonised rules, secure processing environments, interoperable standards, and a federated architecture connecting health data holders, HDABs, and HealthData@EU. These lessons provide the foundation for the conceptual model proposed in the following section, which illustrates how EHDS-enabled infrastructures could support the secure, federated, and interoperable transformation of distributed health data into actionable intelligence for surveillance and preparedness.

## 4 Recommendations and options for an EHDS-enabled European public health surveillance framework

The previous sections showed that Europe already has many of the data sources, institutions, and surveillance capacities needed to strengthen public health surveillance and emergency preparedness. However, these elements remain distributed across different health systems, institutions, governance structures, and technical infrastructures, limiting the ability to generate timely and comparable intelligence for public health surveillance. As explored in Section 3, the main limitation is therefore not the absence of data or expertise, but the lack of an integrated governance and interoperability layer capable of transforming fragmented health information into an integrated ecosystem.

Therefore, we propose a conceptual model in which the EHDS functions as the enabling layer that connects distributed health data sources with existing public health surveillance capacities and regulatory systems. This model follows the basic steps that define an effective surveillance system: data collection, analysis, interpretation and response, which helps to inform and support public health action (27). Figure 3 presents the proposed European public health intelligence ecosystem, organised around three complementary layers in a bottom-up data-to-action flow: distributed health data sources; the EHDS enabling layer; and public health intelligence and decision-making.

**Figure 3:**
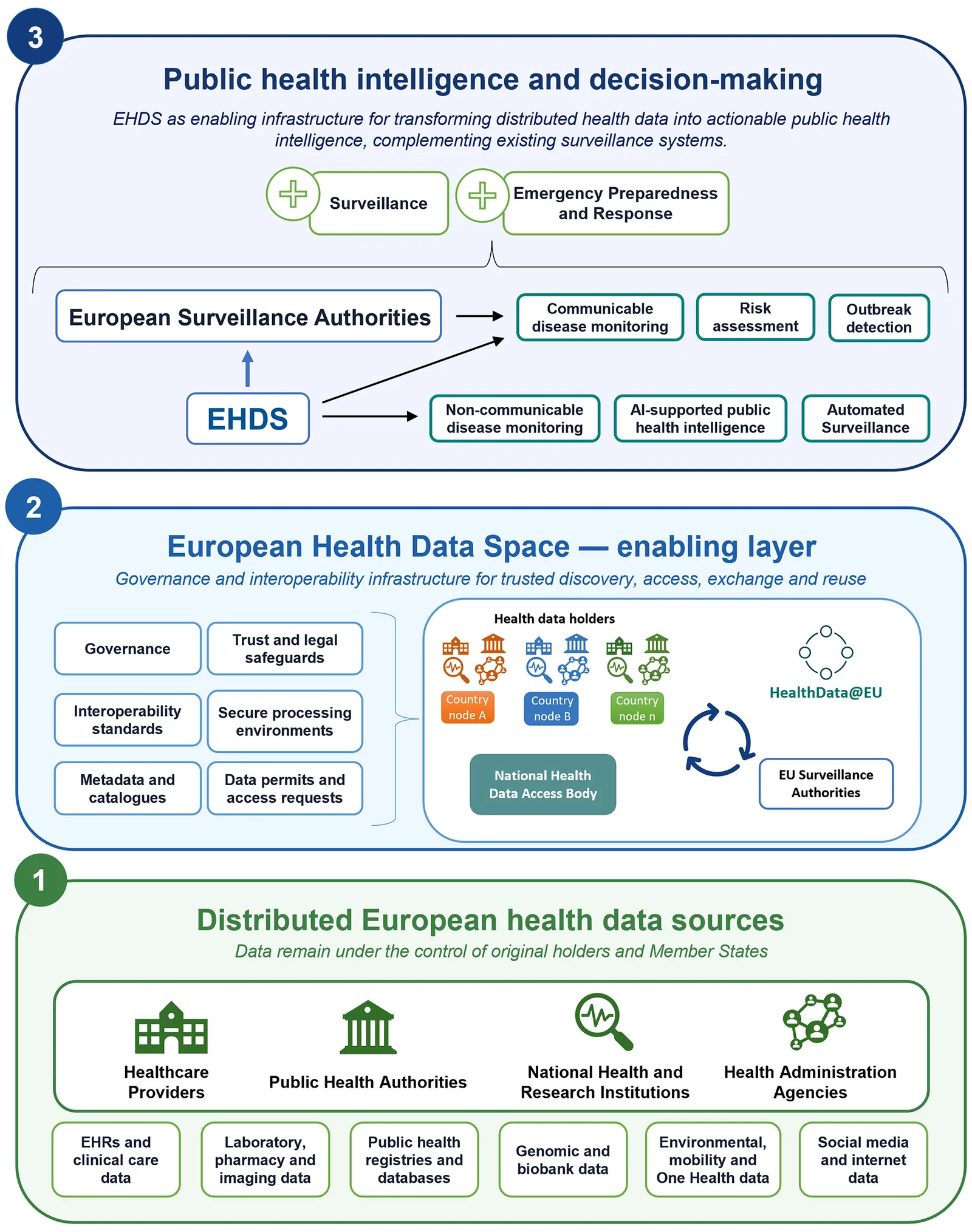
Scheme illustrating the proposed conceptual EHDS-enabled European public health intelligence ecosystem. The complementary layers represent a bottom-up flow of health data for enhanced public health surveillance and emergency preparedness and response: (1) distributed European health data sources layer, (2) the EHDS enabling layer, and (3) public health intelligence and decision-making layer.

At the foundation of the ecosystem are health data holders, which retain control over their respective datasets in line with the principles of subsidiarity, national responsibility, and data sovereignty that characterise European health systems. The second layer represents the EHDS enabling layer, which provides the governance rules, interoperability standards, the institutional federated system for the secure and legally compliant secondary use of health data. This framework is operationalised by the communication between data holders, HDABs, the HealthData@EU and EU surveillance authorities, that allow data sharing through secure processing environments, data permits, and authorised participation mechanisms. The third layer corresponds to public health intelligence and action, where data accessed and reused through the EHDS enabling layer are analysed and transformed into a continuous intelligence for surveillance. Surveillance-relevant outputs are then interpreted by epidemiologists, clinicians, statisticians, public health professionals, regulators, and decision-makers, and connected to the existing European surveillance and regulatory authorities controlled by EU agencies. In this model, the added value of the EHDS lies in complementing these systems by providing access to broader, richer, and more continuous data flows, supporting established functions such as communicable disease monitoring, risk assessment, and outbreak detection, while also enabling expanded capabilities such as non-communicable disease monitoring, AI-supported public health intelligence, and automated surveillance. Therefore, the EHDS can strengthen surveillance and preparedness capabilities at the EU, allowing timely intelligence gathering and a more coordinated evidence-informed decision-making across Member States.

### 4.1 Operationalising the European public health intelligence ecosystem: organisational governance options under the EHDS

This conceptual model could be operationalised by considering how distributed data from different sources could be connected under the EHDS framework to support public health surveillance and emergency preparedness. Operationalising this vision requires appropriate organisational mechanisms capable of connecting health data holders, HDABs, public health authorities, the European HealthData@EU infrastructure, and EU-level public health surveillance bodies. To identify these mechanisms, the organisational provisions of the EHDS Regulation that could provide legal and institutional foundations for establishing these links were analysed. Provisions concerning authorised participants in HealthData@EU, health data intermediation entities, and the designation of one or more HDABs offer different pathways through which an EHDS-enabled public health intelligence ecosystem could be organised and strengthened.

Building on these provisions, three conceptual governance options for how health data could be accessed, shared, and analysed for public health surveillance and preparedness through federated and decentralised approaches are proposed (Figure 4). These governance options are derived from organisational possibilities described in the EHDS Regulation and do not represent specific implementation models prescribed by it. Rather, they illustrate possible ways of adding an EHDS operational layer to the existing European public health surveillance systems, while preserving data protection, institutional trust, and cross-border interoperability. The governance options are non-exclusive and differ mainly in the entity responsible for coordinating surveillance-related data access and processing within the EHDS operational layer, the degree of centralisation or federation, and the relationship between national infrastructures and HealthData@EU. In this way, different surveillance authorities involved in public health surveillance could benefit from different organisational options. As such, each surveillance institution can leverage the EHDS according to their roles, responsibilities, and data needs, supporting public health intelligence and surveillance at both national and European levels.

**Figure 4:**
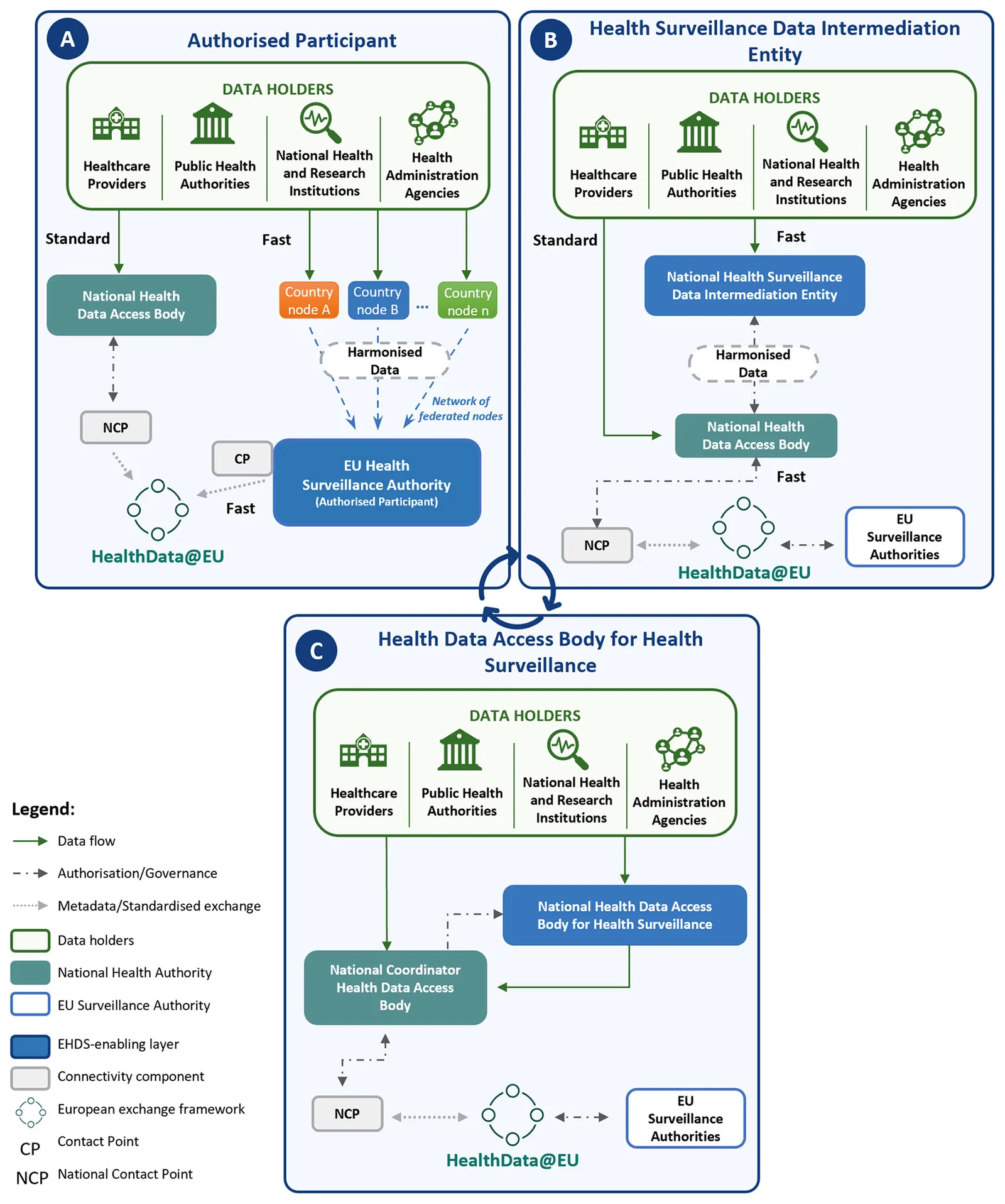
Conceptual governance structures for enabling public health surveillance within the EHDS framework. Three alternative organisational governance options are proposed, based on different provisions of the EHDS Regulation for connecting health surveillance data holders, national HDAB, national contact points (connectivity component), and HealthData@EU: **(A)** an Authorised Entity Platform, **(B)** a Health Surveillance Data Intermediation Entity, and **(C)** a dedicated HDSB for public health surveillance. A legend of the icons meaning is provided in the bottom left of the Figure.

***Option A—European Health Surveillance Bodies as Authorised Participants in*** ***HealthData@EU***

Option A explores the possibility of establishing a European health surveillance platform as an authorised participant in the HealthData@EU under Article 75 of the EHDS Regulation (Figure 4A). This article allows for health-related research infrastructures or similar infrastructures established in the EU to become authorised participants connected to the HealthData@EU. Under this model, the authorised participant would connect to HealthData@EU and function as a European-level surveillance authority dedicated to supporting cross-border public health surveillance, preparedness, and response. Under this model, EU public health bodies such as ECDC, HERA, EMA and others could become authorised participants and connect directly to Healthdata@EU, having access to richer and more diverse health data sources.

In this option, health data would remain distributed across national data holders but compliant with defined common data standards. The proposed European health surveillance authorised participant would be dedicated to epidemiological surveillance functions and connect to HealthData@EU through its contact point. Furthermore, as an authorised participant, the platform could assess applications and issue access approvals for data falling within its remit, which would expedite decisions on access to surveillance data. Access to other national datasets would continue to follow the EHDS governance framework through national HDABs.

The role of the European health surveillance authorised participant would therefore be to coordinate surveillance-related data applications, accelerating epidemiological data collection, preparation and analysis and facilitating the aggregation and comparison of surveillance indicators across Member States. Rather than requiring the creation of a central European health data repository, this entity could rely on a network of federated nodes, similar to the infrastructure under EUCAIM (44), allowing data to remain within national infrastructures while enabling analytical activities at the European level. Furthermore, separate health surveillance authorised participants could be established for different specific surveillance domains, following the example of a federated model being implemented by Spain for the secondary use of health data (57).

The main advantage of this option is its potential to establish a European health surveillance platform capable of supporting multinational data analyses and coordinated responses to cross-border health threats at the EU level. Where data fall within its defined remit, this platform could help expedite decisions on cross-border data access and ensure data harmonisation and interoperability across national nodes. This solution offers high scalability and cross-border applicability, but its implementation could require robust governance arrangements, technical investment, coordination with national HDABs and improved interoperability across national nodes.

***Option B—National health surveillance data intermediation entity***

Option B is based on Article 50(3) of the EHDS Regulation, which allows Member States to establish health data intermediation entities responsible for fulfilling certain obligations of health data holders that lack technical and/or personnel resources to perform their duties (Figure 4B).

Under this model, a national health surveillance data intermediation entity would act as a dedicated technical and organisational layer between health data holders and the national HDAB(s), supporting the preparation and availability of relevant data for secondary use in public health surveillance and emergency preparedness. This intermediation entity could provide a dedicated organisational and technical layer for epidemiological surveillance, supporting activities such as data standardisation, quality assessment, metadata management, and the preparation of datasets for secondary use (58). The national HDAB would retain its regulatory and governance responsibilities for managing access to health data, while the intermediation entity would facilitate the operational functions between multiple health data holders and the HDAB.

This model could be particularly relevant for Member States where surveillance-relevant data is highly heterogeneous and fragmented across multiple institutions and information systems. By creating a specialised intermediary infrastructure, this option could reduce the operational burden on individual data holders and improve the availability of harmonised, interoperable, and well-documented national datasets for surveillance purposes. It could also accelerate the preparatory steps required after data access has been approved, increasing the quality and usability of data available for epidemiological analysis, public health intelligence, and preparedness planning.

However, this option would mainly strengthen the national layer of the EHDS ecosystem. Decisions on data access would remain under the responsibility of the relevant HDAB(s), which according to EHDS provisions may take up to 2 months for public health sector bodies and Union institutions. Moreover, cross-border access by EU-level surveillance authorities would still require the submission of data applications via HealthData@EU. Therefore, its contribution to European-level surveillance would depend on the extent to which national intermediation entities are implemented consistently across Member States and aligned with common interoperability standards. This model could improve national readiness but have a more limited impact on the scalability and creation of a coordinated European public health intelligence ecosystem.

***Option C—Dedicated health data access body for public health intelligence***

Option C is based on the provisions of Articles 55, 57, 58, 59, 63, and 64 of the EHDS Regulation, concerning the designation, responsibilities, and operation of the HDAB. Article 55, in particular, allows Member States to designate one or more HDABs responsible for implementing the secondary use framework. Under this organisational framework, a Member State could designate a dedicated HDAB responsible for managing access to health data for public health surveillance and intelligence.

This specialised HDAB would operate alongside the national coordinator HDAB responsible for coordinating the broader implementation of the EHDS for the secondary use of health data. The dedicated surveillance HDAB could manage access to health data for surveillance-related purposes, coordinate interaction with relevant data holders, assess surveillance-specific data applications, and facilitate access to data through secure processing environments. The coordinating HDAB would maintain responsibility for ensuring consistency with the national EHDS governance framework and for coordinating the interaction between the HDAB for surveillance and the HealthData@EU infrastructure.

The main advantage of this organisational framework is the establishment of a specialised governance structure with explicit responsibility over public health surveillance and preparedness. This would facilitate the timely exchange of health data and the development of dedicated expertise, procedures and analytical capabilities for the specific requirements of public health surveillance data. This organisational framework could open the way to more expedite decisions on data applications for epidemiological surveillance purposes and provide a clear institutional interface for applying the EHDS secondary-use framework to surveillance-related data requests involving national public health authorities.

However, this option also presents limitations. The designation of multiple HDABs would require strong coordination mechanisms to avoid fragmentation, duplication of responsibilities, and inconsistent interpretation of EHDS provisions. Clear governance arrangements would therefore be needed to define the respective responsibilities of the coordinating HDAB and the surveillance-specific HDAB. In addition, because HDABs are primarily responsible for managing data access procedures rather than preparing or harmonising datasets, this model may provide less technical support for data preparation and interoperability than Options A or B, unless complemented by dedicated technical infrastructures or intermediation entities. Like Option B, cross-border access by EU-level surveillance authorities would still require applications through HealthData@EU.

Overall, these three governance options illustrate different ways in which the organisational mechanisms foreseen in the EHDS Regulation could be used to operationalise a European public health intelligence ecosystem. These governance options therefore provide a conceptual basis for discussing how the EHDS could support the transformation of distributed health data into actionable intelligence for surveillance, and emergency preparedness and response. A comparative assessment of the opportunities and limitations of each option is provided in Section 5, sub-section 5.3 and in Table 1.

**Table 1:** Comparative assessment of the organisational framework options.

Operational framework option	A	B	C
Primary purpose	**Centralised EU platform acting as Authorised Participant**	**National Data Intermediation Entity for surveillance**	**Dedicated HDAB for surveillance**
EHDS-related articles	**75**	**50**	**55, 57, 58, 59, 63, 64**
Advantages	- Enables coordinated EU-wide surveillance	- Provides a specialised intermediary for surveillance data	- Establishes explicit institutional responsibility for surveillance
	- Supports federated and cross-border analyses	- Reduces operational burden on individual data holders	- Provides specialised governance and technical expertise
	- Promotes data harmonisation across Member States	- Facilitates data preparation and harmonisation	- Supports national surveillance activities
	- High potential for scalability	- Adaptable to national health systems infrastructures	- Expedites decisions on access to data
	- Facilitates expedited coordinated responses to cross-border health threats	–	–
Disadvantages	- High implementation and governance complexity	- Introduces an additional organisational layer	- Requires clear governance mechanisms to avoid duplication of responsibilities
	- Requires substantial EU-level coordination	- Requires clear allocation of responsibilities	- May require additional institutional and technical resources
	- Requires interoperability across heterogeneous national infrastructures	- Limited contribution to EU-wide coordination	- Does not enforce data harmonisation neither expedites data preparation
	- Requires clear governance arrangements with national HDABs	- Implementation may vary considerably between Member States	- Implementation models may differ between Member States

Bold values refer to the provision articles of the EHDS Regulation.

## 5 Discussion

Much of the existing literature on the EHDS has focused on its role in enabling the secondary use of health data for research and innovation, with less attention given to its potential contribution to public health surveillance and emergency preparedness, despite being among the purposes for secondary use explicitly recognised in the EHDS Regulation (17, 19). This represents an important gap, as effective preparedness depends not only on the availability of relevant data, but also on the capacity to transform heterogeneous data into timely, interpretable, and actionable public health intelligence. This study draws attention to this underexplored dimension of the EHDS: its potential strategic value for strengthening public health surveillance and emergency preparedness in Europe.

Through a literature and regulatory-based analysis, we demonstrated that health system digitalisation, EHR implementation and the COVID-19 pandemic have reshaped surveillance and preparedness needs in the EU. Our findings suggest that digital public health tools, routinely collected EHR data and non-traditional data sources can substantially strengthen public health surveillance and emergency preparedness by supporting disease monitoring, earlier threat detection, real-world evidence generation, and more targeted public health action and evidence-informed decision-making (8, 13, 33, 35). However, this potential remains limited by persistent structural barriers, including fragmented information systems, uneven digital maturity, variable data quality, limited interoperability, difficulties in EHR implementation for secondary uses, and governance and legal uncertainty across Europe (12, 23–25). Europe's challenge is no longer to generate sufficient electronic health data, but to make existing data usable, interoperable, trustworthy, and actionable for public health purposes. The COVID-19 pandemic made these limitations particularly visible, while also accelerating the recognition that surveillance and preparedness depend on common standards, comparable data, decentralised or federated data-sharing approaches, and trusted governance mechanisms (25, 30–37). As such, improving the use and exchange of health data for surveillance, preparedness and emergencies response requires more than technological progress. It depends on ethical, legal, organisational, and political frameworks capable of enabling trusted and timely data sharing across institutions and Member States to generate public health intelligence and improve evidence-informed decision-making.

Our regulatory analysis indicated that the EHDS provides important foundations for addressing some of the persisting challenges identified in the literature review and the fragmentation that currently characterises health data ecosystems across Member States. By defining permitted purposes for secondary use, relevant data categories, data holder responsibilities, HDAB functions, secure processing environments, and cross-border access through HealthData@EU, the EHDS Regulation creates the conditions for a more coordinated, transparent, and legally supervised health data exchange ecosystem, providing a common framework through which health data can become more findable, accessible, interoperable, and reusable for public health purposes. Therefore, the EHDS can be conceptualised as an important enabling layer that connects distributed health data sources, public health authorities, existing surveillance infrastructures, and European bodies into a coordinated public health intelligence ecosystem. Rather than proposing an extension of the Regulation's legal scope, this perspective highlights how the capabilities and organisational arrangements established under the Regulation could be leveraged to strengthen existing public health surveillance and preparedness systems at national and European levels.

### 5.1 Integrating the EHDS with existing surveillance and preparedness systems

This study does not position the EHDS as a replacement for established surveillance systems, national public health infrastructures, or EU-level bodies such as ECDC, EMA, and HERA. These actors remain responsible for their respective surveillance mandates, epidemiological interpretation, risk assessment, emergency coordination, and public health response (6, 7, 59). The European Surveillance System (TESSy) remains essential for communicable disease monitoring and outbreak detection (59). Similarly, the evolution of the Early Warning and Response System (EWRS), a communication platform for the alerting and risk management of serious cross-border threats to health, has strengthened coordinated action across the EU (60). These surveillance mechanisms will likewise continue to play a central role in communicable disease monitoring and outbreak detection but they are organised around disease-specific reporting, predefined datasets, and national submission processes, limiting integration with broader health data sources and longitudinal population-scale analyses (59). As such, the ECDC recognised the need for a more digitalised and integrated EU/EEA surveillance system capable of connecting with other data sources, digital platforms, and the EHDS (6).

The relevance of the EHDS lies in its potential to complement existing European surveillance and regulatory systems, connecting them with broader and more interoperable sources of health data and supporting the objectives of the European Health Union (17, 61). Its added value is therefore not limited to facilitating data access, but extends to enabling more secure, harmonised, and trusted secondary use of health data for public interest purposes, including surveillance, preparedness, research, policymaking, and regulatory activities, reducing the prevalence of “data silos” (17).

### 5.2 Harnessing the EHDS for public health surveillance and emergency preparedness and response: opportunities and challenges

The EHDS impact in public health surveillance and emergency preparedness will depend on its effective and consistent implementation across Europe. By making Member States' integration into HealthData@EU mandatory, legal and infrastructural basis for more coordinated data governance across Member States are provided, strengthening European collaboration (17, 48). However, even though several national e-health infrastructures are preparing to implement the EHDS to enhance the use of health data at the multinational level, many others might require more time to establish the necessary connections. This uneven readiness highlights the importance of addressing differences in national digital maturity, infrastructure, cybersecurity, and digital health literacy to ensure that EHDS implementation delivers equitable benefits across Member States (62). In this context, investment in HDAB capacity, HealthData@EU operationalisation, digital maturity, data quality, semantic interoperability, and technical expertise should not be regarded only as technical implementation requirements, but also as strategic investments in Europe's resilience and preparedness to future health threats.

Federated and decentralised approaches are central to the EHDS vision, supporting cross-border data exchange and analysis while allowing data to remain within national or institutional environments. This may help preserve data sovereignty, reduce the risks associated with large-scale centralisation, and improve resilience of European infrastructures (40). Centralised systems may be vulnerable to system-wide outages, whereas federated personal health data spaces offer greater resilience by distributing data across devices, encrypted backups, or trusted data fiduciaries, but also require robust mechanisms for data integrity, traceability, and auditability across distributed sources, as well as initiatives to strengthen digital and health literacy (40). Clear advantages of a decentralised approach can be achieved through the use of federated learning, which uses a central coordinator to aggregate model updates from multiple sites into a shared global model, and swarm learning, which enables peer-to-peer training without one, supporting cross-border health data analysis (49). However, challenges related to data quality, availability and interoperability affect both centralised and federated systems (40). In addition, decentralised approaches remain at an early stage of development and require scalable infrastructures, data standardisation, and trusted collaboration between HDABs and EU emergency agencies to be used to its fullest (34). Evidence from the path towards EHDS implementation in Italy also suggested that decentralised health systems may face difficulties due to regional fragmentation, uneven digital maturity, and limited semantic interoperability, unless supported by stronger governance, stakeholder engagement, and legal clarity (50). As such, the broader implementation of federated and other decentralised approaches across Europe will require sustained investment in both legislative frameworks and technological infrastructure to enable their effective and secure adoption.

Beyond its important role in infectious disease surveillance, the EHDS could also be leveraged to support broader and more continuous forms of public health intelligence (27). For example, timely access to high-quality, non-aggregated data is extremely relevant for prevention-oriented monitoring of non-communicable diseases, where longitudinal and population-level data can support earlier identification of risks, policy-relevant indicators, and targeted intervention (51). Similarly, initiatives such as the Collaborative Health Information European Framework (CHIEF) further illustrate how common health information systems, citizen participation, real-world data use, pilot projects, and federated data catalogues with searchable metadata could support continuous NCD monitoring and complement EHDS implementation (52). The EHDS could also connect with other European data spaces and contribute to the One Health approach that proposes that human health, animal health, and the environment are all innately interconnected (53). Because human health, animal health, and environmental conditions are interconnected, public health intelligence increasingly depends on the ability to combine health data with environmental, agricultural, and other cross-sectoral data sources (53). A transversal One Health layer across the EHDS, the Agriculture Data Space, and the Green Deal Data Space could improve access to FAIR, quality-controlled, and transdisciplinary data for better zoonotic risk assessment, policymaking, and epidemic preparedness (54). Finally, the EHDS may help create the conditions for more responsible AI-supported public health intelligence. AI tools have the potential to support automated data processing, predictive surveillance, anomaly detection, outbreak modelling, and resource planning, but their value depends on high-quality, interoperable, representative, and well-governed data ecosystems (55, 56). However, AI tools still face significant barriers to widespread implementation in public health surveillance. Poor data quality and limited interoperability can affect the reliability and performance of AI models across different settings, while underdeveloped evaluation metrics make it difficult to compare results across studies and evidence of their real-world impact remains limited (63, 64). At the same time, governance challenges related to data privacy, transparency, accountability, and ethics further require robust regulatory and oversight frameworks, including appropriate regulatory alignment and ethical safeguards, bias monitoring, improved explainability, privacy protection, and human oversight (56). Therefore, although AI has considerable potential to strengthen public health surveillance, its responsible use requires a cautious and proportionate regulatory approach, supported by appropriate legal, ethical, and governance frameworks, including the AI Act, and coordinated phased implementation across Member States (65).

A central implication of our findings is that public health surveillance and emergency preparedness require operational conditions that differ from many other secondary-use purposes. Indeed, article 71(4) recognises that, by way of exception, personal electronic health data subject to an opt-out may still be processed for certain public health purposes, including protection against serious cross-border threats to health and public health surveillance, when the request is submitted by a public sector body or Union institution and the data cannot be obtained by alternative means in a timely and effective manner. Crisis response also requires timely access to comparable, high-quality data when evidence is needed to assess emerging risks, monitor outbreaks, evaluate medical countermeasures, allocate resources, or coordinate responses to cross-border health threats. Several provisions of the EHDS Regulation seek to expedite access to health data for public health surveillance, emergency preparedness, and responses to health threats (17). However, the envisaged access timelines remain on the scale of months rather than the days or hours often required for timely public health action during emergencies. Article 68(6) shortens the HDAB decision period to 2 months, extendable by one additional month, while article 60(2) further provides that health data holders may have up to 3 months to make the requested data available to the HDAB, extendable by an additional 3 months in justified cases.

Article 68(7) provides that the HDAB should make data available within 2 months after receiving them from health data holders, unless a longer timeframe is specified. These provisions establish an important legal basis for the use of health data in emergency preparedness and response, but do not, in themselves, provide a clearly defined operational pathway for rapid data access in situations of crisis. The model proposed in this study helps clarify how such a pathway could be operationalised by connecting the relevant components of the EHDS ecosystem to support faster crisis-response data flows. Under this approach, national health surveillance data intermediation entities could support data preparation and harmonisation (Figure 4B), while dedicated surveillance HDABs could apply prioritised access procedures for applications related to public health surveillance and emerging health threats (Figure 4C). Alternatively, a European health surveillance authority acting as an authorised participant in HealthData@EU could coordinate federated analyses and potentiate faster comparative surveillance intelligence across Member States (Figure 4A).

Therefore, while the EHDS provides an important legal basis for urgent public health data use, additional crisis-ready procedures may be needed to ensure that access decisions, data preparation, and data availability occur within timeframes compatible with emergency response. These procedures could be specified within the EHDS framework, leveraging the secondary-use infrastructure already being developed to define a faster route for emergency scenarios. The future EHDS implementation guides could support this by establishing clearer operational timelines for data access applications, data preparation by health data holders, and data availability in secure processing environments when requests are submitted for crisis-response purposes. For this reason, surveillance and emergency preparedness should be treated as priority use cases in future EU initiatives to test whether accelerated access procedures are sufficiently clear, feasible, and operational before crises occur.

Overall, these findings suggest that the EHDS should be implemented not only as a general secondary-use framework, but also as an infrastructure capable of supporting both long-term surveillance and timely crisis response. If these conditions are met, the EHDS could support a broader public health intelligence ecosystem that supports NCD monitoring, One Health surveillance, responsible AI adoption and a timelier and more effective public health surveillance, emergency preparedness and response. Building on this, the proposed conceptual model and governance options presented in this study help explain how the EHDS enabling layer can be operationalised.

### 5.3 The European public health intelligence ecosystem: a comparative assessment of the organisational governance options

Building on the conceptual model and the three governance options outlined in Section 4, we comparatively assessed the main strengths and limitations associated with each option. The assessment indicates that the three options differ in terms of their national applicability, cross-border scalability, governance complexity, interoperability requirements, and alignment with existing institutional structures. No single model is therefore likely to be optimal across all settings. The suitability of each governance approach will depend on the intended scope and objectives of the surveillance activities, the institutional and legal context of individual Member States, existing digital and governance capacities and the degree of cross-border coordination required.

The main advantages and disadvantages of each option are summarised in Table 1. Option A offers the strongest European-level coordination capacity by enabling an authorised participant in HealthData@EU to support cross-border surveillance intelligence gathering and improve coordinated responses to health threats across Europe. This option offers the greatest potential for EU-wide coordination and scalability, but would require substantial investment in governance, interoperability, technical development, and legal clarity regarding the role and remit of the authorised participant. Option B strengthens the national technical layer by supporting data holders in preparing, harmonising, and making surveillance-relevant data available. Compared with Option A, this option may be more readily integrated into existing national health systems, as it builds on national structures and institutions. However, its potential to support coordinated European surveillance would be comparatively more limited and would depend on consistent implementation across Member States and alignment with common EHDS standards. Option C reinforces the national governance layer by creating a specialised HDAB responsible for surveillance-related data access procedures and improve coordination with public health authorities. This option occupies an intermediate position by combining national specialisation with integration into the broader EHDS governance and cross-border infrastructure. However, it would remain affected by the access timelines established under the EHDS Regulation, if additional crisis-ready procedures are not developed. Even under accelerated procedures for public-sector bodies and Union institutions with a legal mandate in public health, the process from data application to effective data availability may still take several months (66).

As such, the three options should not be understood as mutually exclusive. A more effective model may combine strengths from different options. For example, a hybrid organisational framework could combine the national specialisation through a dedicated surveillance HDAB proposed under option C with the European-level coordination and federated analytical capabilities of an authorised participant in HealthData@EU, as proposed in option A. Under this approach, the national surveillance HDAB could coordinate access to health data for surveillance activities within each Member State, while the European health surveillance platform, operating as an authorised participant in HealthData@EU, could facilitate cross-border analyses, comparative assessments, data harmonisation and coordinated responses to European health threats. Alternatively, combining the functions of a national health surveillance data intermediation entity proposed in options B with option C could strengthen the national layer by promoting data harmonisation and expedite data access decisions. As such, crisis-response may require a combination of the three options, to ensure that access decisions, data preparation, and data availability occur within timeframes compatible with emergency response.

Overall, this comparative assessment suggests that the EHDS should not be viewed only as a mechanism for facilitating access to health data, but as an enabling layer that connects dispersed health data with existing national and European systems, improving data flow, interoperability, and cooperation across Europe. The combination of these options could help address several of the challenges identified throughout Section 3, particularly regarding data fragmentation and the need for timely, interoperable, and coordinated public health surveillance and emergency preparedness at European level. The establishment of clear responsibilities, interoperable infrastructures, trusted governance, and sufficient capacity across Member States will be essential to translate this operational value into practice.

### 5.4 Strengths, limitations, and future directions

This study integrates scientific literature, policy developments, implementation examples, use cases, and regulatory analysis to examine the role of the EHDS in public health surveillance and emergency preparedness. This multi-source approach strengthens the study by providing a comprehensive and policy-relevant perspective that culminated in the proposal of a conceptual model for an EHDS-enabled European public health intelligence ecosystem. Three non-exclusive governance organisational options provided a clear recommendation on how this vision could be made operational using the possibilities described in the EHDS Regulation. However, the proposed governance framework does not represent a technical implementation or a software specification. It provides an evidence-informed framework illustrating how the governance, interoperability mechanisms, and secondary use provisions established in the EHDS could be operationalised to support early threat detection, preparedness planning, and coordinated responses to future cross-border health threats. At the same time, the proposed framework has not yet been empirically validated, and its feasibility will depend on the progressive operationalisation of the EHDS, the maturity of HealthData@EU, the development and capacity of national HDABs, and the adoption of common interoperability standards across Member States.

Therefore, while the reviewed literature and regulatory analysis suggest that the EHDS could play an important role in public health surveillance, emergency preparedness, and crisis response, further empirical work will be needed to assess whether the proposed model can function in practice across different national and European settings. Future work should focus on moving from conceptual design to implementation testing, including stakeholder validation of the proposed model, governance assessments of the three organisational options by relevant EU and national authorities, and data flow testing through regulatory sandboxes, simulation exercises, and pilot projects using real or realistic public health surveillance and emergency-response scenarios. These approaches would allow Member States and EU institutions to evaluate the feasibility and added value of the EHDS enabling layer before full-scale deployment.

In parallel, it is also important to develop the indicators capable of assessing the role of the EHDS in strengthening public health intelligence. The evaluation should not focus only on the number of data access applications, permits issued, or datasets made available, but also on whether EHDS-enabled data use improves the timeliness, comparability, quality, interpretability, and public health relevance of evidence generated for surveillance, preparedness, and response. Future studies should also explore how the EHDS can support broader public health intelligence use cases, including non-communicable disease monitoring, One Health surveillance, responsible AI-supported automated surveillance and accelerate the operational timelines for data access, preparation, and availability during emergencies.

This study highlights the EHDS as an important Regulatory framework to modernise European public health surveillance and strengthen emergency preparedness. Ultimately, the transition from fragmented health data systems to a European public health intelligence ecosystem will depend on the capacity of Member States and EU authorities to collaborate and operate as a coherent whole to protect population health. Future research, EU-funded projects, and European Commission initiatives will be essential to test, validate, and translate this vision into practice.
